# Long, Processive Enzymatic Dna Synthesis Using 100% Dye-Labeled Terminal Phosphate-Linked Nucleotides

**DOI:** 10.1080/15257770802260741

**Published:** 2008-08-18

**Authors:** Jonas Korlach, Arek Bibillo, Jeffrey Wegener, Paul Peluso, Thang T. Pham, Insil Park, Sonya Clark, Geoff A. Otto, Stephen W. Turner

**Affiliations:** Pacific Biosciences Inc., Menlo Park, California, USA

**Keywords:** DNA, dNTP, fluorescence, sequencing applications

## Abstract

We demonstrate the efficient synthesis of DNA with complete replacement of the four deoxyribonucleoside triphosphate (dNTP) substrates with nucleotides carrying fluorescent labels. A different, spectrally separable fluorescent dye suitable for single molecule fluorescence detection was conjugated to each of the four dNTPs via linkage to the terminal phosphate. Using these modified nucleotides, DNA synthesis by ø29 DNA polymerase was observed to be processive for products thousands of bases in length, with labeled nucleotide affinities and DNA polymerization rates approaching unmodified dNTP levels. Results presented here show the compatibility of these nucleotides for single-molecule, real-time DNA sequencing applications.

## Introduction

Nucleotides carrying fluorescent labels are widely used in biological research, molecular biotechnology, and cytogenetics. Applications have reached single-molecule detection capabilities, among them new DNA sequencing technologies based on the detection of individual labeled nucleotides upon enzymatic incorporation into DNA.[1,2] Complete replacement of every deoxyribonucleotide triphosphate (dNTP) by a dye-labeled dNTP is essential to these methods to avoid the creation of sequence gaps. Several of these sequencing-by-synthesis schemes utilize nucleotides with fluorescent dyes linked to the nucleobases, but their enzymatic incorporation becomes increasingly limited with larger fractions of labeled dNTP replacements.[3–7] Solutions involving step-wise additions of base-labeled nucleotides, followed by chemical or photochemical removal of the label, result in reduced sequencing speeds as additional washing and cleavage steps have to be performed.[8,9]

In an alternative approach, the fluorescence label is attached to the terminal phosphate. DNA polymerases induce cleavage of the α-β-phosphoryl bond upon incorporation of a nucleotide into DNA, releasing the pyrophosphate leaving group and attached fluorescent label. Natural DNA is produced, eliminating steric hindrance as a potential cause of enzyme inhibition. Improved fidelity of HIV reverse transcriptase has been reported in conjunction with terminal phosphate-labeled nucleotides.[10] A homogeneous assay for single nucleotide polymorphism detection has also been described.[11] In this assay, single nucleotide incorporations were sufficient. An extension of the triphosphate moiety to four and five phosphates was reported to increase incorporation efficiencies. Using a large excess of polymerase, complete replacement of all dNTPs by terminal phosphate linked dNTPs was shown, reaching as much as 20 bases on a linear template.[12] It was not determined whether DNA synthesis was processive under the reaction conditions used.

The efficient utilization of dye-labeled nucleotides by DNA polymerases constitutes an important prerequisite for single molecule DNA sequencing-by-synthesis technologies. Here, we demonstrate processive enzymatic DNA synthesis, thousands of nucleotide incorporations in length, using exclusively terminal phosphate-labeled nucleotides. Unique fluorophores, suitable for single molecule detection and differentiated by their emission spectra, were used for each of the four nucleobases. The kinetic properties of DNA polymerization were similar to those for unmodified dNTPs.

## Synthesis and Results

**SCHEME 1:**
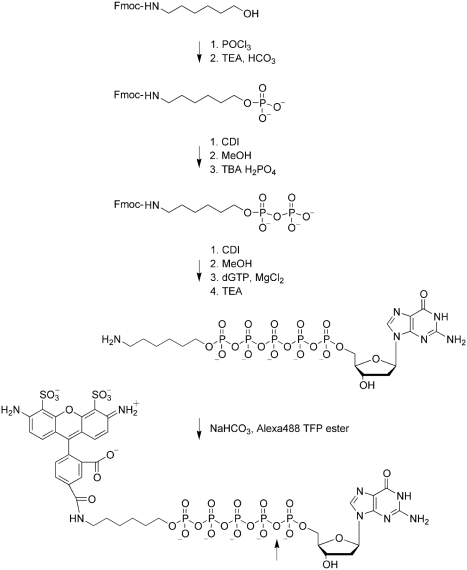
Chemical synthesis of terminal phosphate linked nucleotides, shown by example for Alexa Fluor 488 aminohexyl-O-dG5P (A488-dG5P, described in detail in Methods). The arrow depicts the cleavage site by the enzymatic activity of DNA polymerase, separating the fluorophore from the nucleotide during incorporation into DNA.

The chemical synthesis of the terminal pentaphosphate-labeled nucleotides (dN5Ps) is shown in Scheme 1, using Alexa Fluor 488 aminohexylO-dG5P (A488-dG5P) as an example. Original synthesis routes for *triphosphate*-linked nucleotides, employing cyclization of the triphosphate followed by nucleophilic attack of a primary amine on the phosphate ring,[13] do not extend well to compounds containing longer phosphate linkers because of the lower nucleophile strength of phosphates compared to primary amines. The synthesis route used in this study was adapted from a procedure described by Sood et al.,[11] using carbonyldiimidazole (CDI) activation. It was modified by building the pyrophosphate moiety on a linker instead of directly on the dye, allowing for dye conjugation in the final step. The aliphatic linker also allows larger spatial separation between nucleotide and fluorophore. Secondly, MgCl_2_ was included during the second CDI activation for linker-pyrophosphate to dNTP coupling which significantly improved the yield of this synthesis step.[14] In contrast to base-linked nucleotide derivatizations, the chemical synthesis scheme employed here proceeds identically for all four nucleobases.

DNA polymerase from bacteriophage ø29 was used to investigate the enzymatic compatibility with these modified nucleotides. ø29 DNA polymerase harbors favorable properties for single molecule DNA sequencing applications, such as a high processivity up to several hundred kilobases, maximum synthesis rates of ∼100 bases/s, and a low error rate of ∼10^−.5.^[15,16] It is also capable of strand displacement DNA synthesis, enabling the use of double stranded DNA as templates.[17]

**FIGURE 1:**
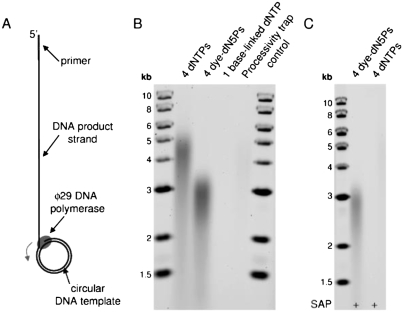
Long, processive DNA synthesis using complete replacement of all four unmodified dNTPs by fluorescent, terminal phosphate-labeled nucleotides. (A) Principle of rolling-circle, strand-displacement DNA synthesis by ø29 DNA polymerase. (B) DNA synthesis products after 5 minute extensions. Reaction conditions are noted above each lane. For one base-linked dNTP, dCTP was replaced by Alexa Fluor 488-7-OBEA-dCTP. In the processivity trap control, the excess oligonucleotide trap was added before the polymerase during the polymerase/DNA template binding step. (C) DNA synthesis including shrimp alkaline phosphatase (SAP) to rule out DNA synthesis by unmodified dNTP contaminants.

Primer extension assays were performed using a small, circular DNA template to permit unlimited, rolling circle DNA strand displacement synthesis (Figure 1A). Long and processive DNA synthesis after complete replacement of all four unmodified dNTPs by the labeled dN5Ps is shown in Figure 1B. The DNA reached an average length of ∼3000 bases after 5 minutes (∼10 bases/second), compared to ∼4500 bases (∼15 bases/second) with unmodified dNTPs. Longer extension reactions resulted in longer DNA products (data not shown; > 10,000 bases after 20 minutes, beyond the limit of the molecular DNA size marker employed). Agarose gel band intensities were similar compared to the control reaction, indicating unaltered DNA polymerization initiation properties. In contrast, no detectable DNA product was formed when using even just one base-linked dye-labeled nucleotide alongside three unmodified dNTPs (Alexa Fluor 488-7-OBEAdCTP instead of dCTP).

In each reaction, a large excess of an oligonucleotide trap was added with the start of the primer extension reaction, ensuring that each DNA strand was synthesized by a single polymerase in a continuous, processive manner. Polymerases dissociating from the primed DNA template during DNA polymerization bind to the trap, removing them from further DNA synthesis. A control reaction demonstrating the effectiveness of the trap is shown in Figure 1B, whereby DNA synthesis was drastically suppressed by including excess oligonucleotide in the DNA template solution *before* the polymerase was added.

To rule out that the observed DNA synthesis could have resulted from traces of unmodified dNTP contaminants, control reactions with shrimp alkaline phosphatase (SAP) were carried out (Figure 1C). Unmodified dNTPs are degraded to the inactive nucleoside by the phosphatase while terminal phosphate-labeled dNTPs are protected by the fluorophore and remain intact.[12,18] This was confirmed for the modified dN5Ps used in this study, yielding undetectable dNTP levels by thin-layer chromatography and HPLC (<0.1%, data not shown). DNA synthesis with four labeled dNTPs was not affected by the inclusion of SAP, demonstrating that the observed DNA synthesis required exclusive utilization of modified nucleotides. In contrast, control reactions using unmodified dNTPs showed no detectable DNA synthesis. This experimental control points toward a beneficial feature of terminal phosphate-labeled nucleotides: Unmodified dNTPs in solution can be enzymatically inactivated by the action of the phosphatase, even during a DNA synthesis reaction. Because the phosphate terminus in base-linked dNTPs is available, they are not compatible with such in situ purification.

**FIGURE 2:**
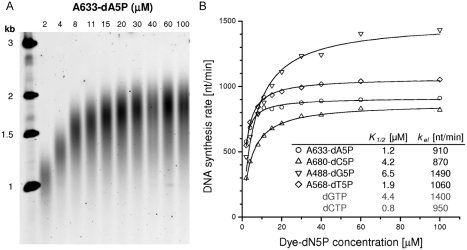
Kinetic characterization of terminal phosphate labeled dN5Ps. (A) Primer extension products from 2 minute DNA synthesis reactions with variable concentrations of A633-dA5P. (B) DNA synthesis rates as a function of concentration for all four modified nucleotides, with fits used to extract kinetic parameters shown in the inset table.

The polymerization kinetics for each analog was determined separately. A quantitative comparison was performed by varying dN5P concentrations in DNA extension reactions to determine apparent substrate specificities (Figure 2). Here, in addition to the labeled dN5P, DNA synthesis encompasses the incorporation of three unmodified dNTPs. Thus the resulting kinetic parameters are only amenable to relative comparisons. An example of increasing DNA lengths with increased substrate concentrations is shown in Figure 2A. The extracted average DNA synthesis rates were plotted against the labeled nucleotide concentrations to obtain maximum DNA synthesis rates, *k_el_*, and characteristic substrate concentrations, *K_1/2_*, at which DNA extension rates reached one half of the maximum synthesis rates (Figure 2B). Values for the four labeled dN5Ps were similar to parameters obtained by this assay for unmodified dNTPs (Figure 2B, inset). The observed small differences in DNA synthesis speeds are at least in part attributable to the inherent differences between the unmodified dNTPs, although contributions from specific dye interactions with the protein surface that could alter the polymerization kinetics cannot be ruled out. This requires more detailed kinetic characterizations that are beyond the scope of this article.

In summary, we demonstrated the efficient synthesis of DNA upon complete replacement of all four dNTPs with nucleotides carrying fluorescent labels at the terminal phosphate. DNA synthesis by ø29 DNA polymerase was processive over thousands of bases, with kinetics approaching that of unmodified dNTPs.

## Methods

### Synthesis of Deoxyribonucleotide Pentaphosphates (dN5Ps)

The synthesis is described using Alexa Fluor 488-aminohexyl-dG5P (A488-dG5P) as an example.

*Fmoc-6-aminohexylphosphate:* Fmoc-6-aminohexanol (1 g, 2.94 mMoles) was co-evaporated with anhydrous acetonitrile (2 × 20ml) then suspended in 10 ml anhydrous triethylphosphate. Phosphorus oxychloride (550 μl, 5.88 mMoles, 2 eq.) was added to the stirring suspension.[19] After 2 hours, HPLC showed disappearance of the Fmoc-aminohexanol. The reaction was quenched by the addition of 100 ml 0.1M triethylamine bicarbonate (pH 6.8) and stirred for 30 minutes. The compound was purified by reverse phase HPLC on a Waters Xterra C18 RP 30×100 column using an acetonitrile gradient in 0.1M triethylamine bicarbonate. The fractions containing product were evaporated, followed by co-evaporation with methanol (2×). The residue was triturated twice with 100 ml diethylether and dried under vacuum to give a white powder. Yield: 1.24 g, 68% as bis-triethylamine salt. HPLC 98%.

*Fmoc-6-aminohexyldiphosphate:* Fmoc-6-aminohexylphosphate (200 mg, 320 µ Moles) was co-evaporated twice with anhydrous acetonitrile, then taken up in 2 ml anhydrous DMF. 1,1′-Carbonyldiimidazole (CDI, 207 mg, 1280 μ Moles, 4 Eq.) was added and stirred at ambient temperature for 4 hours.[20] Methanol (77 μl, 1920 μ Moles) was added and stirred 30 minutes. Tributylamine-H_2_PO_4_ (3200 μ Moles, 10 Eq.), prepared by mixing equimolar amounts of tributylamine and 85% phosphoric acid followed by co-evaporation 3 times with anhydrous acetonitrile, was dissolved in 4 ml anhydrous DMF and added to the reaction. The reaction mixture was stirred 16 hours. HPLC showed 3% Fmoc-aminohexylphosphate remaining. The reaction mixture was diluted to 50 ml with 0.1M TEAB, and was purified by RP HPLC on a Waters Xterra C18 RP 30×100 column using an acetonitrile gradient in 0.1M triethylamine bicarbonate. The fractions containing product were evaporated, followed by co-evaporation with methanol (2×). The residue was co-evaporated with anhydrous acetonitrile. Yield: 186 mg, 73% as tris-TEA salt. HPLC 96%.

*Aminohexyl-dG5P:* Fmoc-6-aminohexyldiphosphate (186 mg, 233 μ Moles) was co-evaporated twice with anhydrous acetonitrile, then taken up in 3ml anhydrous DMF. CDI (150 mg, 930 μMoles, 4 Eq.) was added and stirred at ambient temperature for 4 hours. Methanol (56 μl, 1400 μMoles) was added and stirred 30 minutes. dGTP (TEA salt, 350 μMoles, 1.5 Eq.) was co-evaporated 3 times with anhydrous acetonitrile and suspended in 2 ml anhydrous DMF. The Fmoc-aminohexyldiphosphoimidazolate reaction was added to the dGTP solution followed by anhydrous MgCl_2_ (3500 μMoles, 333 mg, 10 Eq.).[14] The reaction was stirred 18 hours. HPLC showed 28% of the Fmoc-aminohexyldiphosphate converted to Fmoc-aminohexyl-dG5P. The reaction mixture was diluted to 125 ml with 0.1M TEAB, and was purified by RP HPLC on a Waters Xterra C18 RP 30×100 column using an acetonitrile gradient in 0.1M triethylamine bicarbonate. The fractions containing product were evaporated, followed by co-evaporation with methanol (2×). The residue was taken up in 20 ml 10% TEA/water and stirred 16 hours to remove the Fmoc protecting group from the amine on the linker. Triethylamine was evaporated, water was added to 25 ml and the solution was extracted 3 times with 25 ml diethyl ether. The product was purified from the aqueous layer by anion exchange chromatography on Q sepharose FF using a TEAB gradient. Yield 42 μMoles, 18%, HPLC 98%.

*Alexa Fluor 488-aminohexyl-dG5P (A488-dG5P):* Aminohexyl-dG5P (1 μMole) was dissolved in 0.05 M NaHCO_3_ pH 8.7 (200 μl) and was added to 1 mg Alexa Fluor 488-TFP ester (Invitrogen, Carlsbad, CA, USA). The mixture was briefly sonicated. After 4 hours, HPLC showed no active ester remaining. The product was identified by characteristic PDA scan. The compound was purified by IEX on Q sepharose FF with a TEAB gradient. The product was further purified by RP HPLC on a Waters Xterra RP C18 19 × 100 column using an acetonitrile gradient in 0.1M TEAB. The fractions containing pure product were evaporated, followed by co-evaporation with methanol (2×). The residue was dissolved in water and was quantitated by UV-Vis spectrophotometry. Yield 370 nMoles 37%, HPLC 99%.

The other dNTPs were derivatized with Alexa Fluor 633 NHS ester (aminohexyl-dA5P), Alexa Fluor 680 NHS ester (aminohexyl-dC5P), and Alexa Fluor 568 NHS ester (aminohexyl-dT5P).

### DNA Synthesis Assays

Protocols for DNA template and φ29 DNA polymerase generation are given in the Supplementary Information. DNA extension reactions were carried out in a two-step procedure. The first step comprised the formation of the DNA polymerase–DNA template complex. 30 nM of φ29 DNA polymerase (see supporting information) and 100 nM of the primed, circular DNA template were incubated for 5 minutes at room temperature (23°C) in a buffer containing 50 mM Tris-HCl, pH 7.5, 75 mM KCl, 20 mM (NH_4_)_2_SO_4_, and 5 mM DTT. For negative control reactions demonstrating the processivity of the DNA polymerase, a 200-fold molar excess of an oligodeoxynucleotide was included (5′-ACGTTGACAATAACACACTCTATGACACCACCTACCACCTATCTACATC ACC-3′, IDT, Coralville, IA, USA). In such cases, this trap was added before the polymerase. In all other cases, the oligonucleotide trap was added with the extension reaction, after polymerase/template complex formation had occurred.

DNA extension solutions varied according to the specific conditions studied. Each included 6 mM MnCl_2_ as the catalytic metal ion, 40 μM of each dNTP or fluorescently labeled dN5P as applicable, and the DNA “trap” described above. For control reactions involving a base-linked dNTP, ChromaTide Alexa Fluor 488-7-OBEA-dCTP (Invitrogen) was added to 20 μM. Where applicable, shrimp alkaline phosphatase (SAP, USB Corp., Cleveland, OH, USA) was added to a final concentration of 0.075 units/μl and the solution was preincubated for 20 minutes at 30°C to remove any trace concentrations of unmodified dNTPs. In the second step, DNA synthesis reactions were initiated by mixing equal volumes of the template binding and extension reaction solutions (40 μl each). The final concentrations for polymerase, template/primer, DNA trap, MnCl_2_, and nucleotides are reduced by one half because of this mixing procedure. Incubations were performed for 5 minutes at 30°C. Samples of the reaction (20 μl) were quenched with 6 μl of 0.5 M EDTA.

For the kinetic characterization of modified nucleotide incorporation, the polymerase concentration was reduced and DNA extension times shortened to rule out possible artifacts arising from nucleotide depletion during the course of DNA synthesis. The polymerase–template complex formation step was modified to contain 4 nM polymerase and 200 nM circular DNA template/primer. Extension solutions contained 3 mM MnCl_2_, one of the terminally-labeled nucleotides at various concentration (4, 8, 16, 22, 30, 40, 60, 80, 120, or 200 μM, respectively) and three unmodified dNTPs at 20 μM each. Reactions were initiated by mixing equal volumes (20 μl) of the two solutions as described above. After incubation at 30°C for two (Alexa Fluor 633-dA5P, Alexa Fluor 488-dG5P, and Alexa Fluor 568-dT5P) or three minutes (Alexa Fluor 680-dC5P), 30 μl of the reaction was quenched with 9 μl of 0.5 M EDTA.

Reaction products were separated on 0.8% agarose gels, stained with SybrGold (Invitrogen), and imaged on a Typhoon scanner (GE Healthcare). DNA product lengths were extracted using ImageQuant (GE Healthcare) based on the maximum density of DNA band distribution. The validity of the extracted lengths against the double-stranded marker bands was confirmed by comparison to linear single-stranded DNA of known lengths (M13mp18 and X174 single-stranded DNA (NEB, Ipswich, MA, USA), linearized by restriction enzymes).

For the kinetic analysis, average DNA synthesis rates, ν, were extracted for each substrate concentration,*c*, by dividing the obtained lengths by the reaction time. Fitting according to a hyperbolic model yielded values for the maximum DNA synthesis rate, *k_el_*, and the substrate concentration, *K_1/2_*, at which DNA extension rates reached one half of the maximum synthesis rate:

(1) $$\nu =\frac{k_{el}\cdot c}{k_{1/2}+c}$$

## Supporting Information

**DNA template generation.** Oligonucleotides were obtained from Integrated DNA Technologies (IDT, Coralville, IA). The template sequence was 5′-AGATGCAGTCAGTCAGTCAGTCAGTCAGTCAGTCAGTCAGTCAGTCAGTCAGTCAGTCAGTCAGTCACGTCT-3′, harboring of a repeated motif of “agtc” throughout the template (except for the primer binding site where the four base motif was scrambled) to ensure equimolar substrate utilization for all four bases. 15 μM DNA was circularized using 400 units CircLigase (Epicentre Biotechnologies, Madison, WI, USA) in 160 μl reaction solution containing 1 × CircLigase buffer supplied by the manufacturer, 0.1 mM ATP and 2.5 mM MnCl_2_. Ligation reactions were carried out for 60 hours at 60°C. Unligated DNA was removed by addition of 60 units exonuclease I and 600 units of exonuclease III (USB Corp., Cleveland OH, USA), and 10 μl of water, incubation for 30 minutes at 37°C and enzyme inactivation for 15 minutes at 80°C. Primer annealing was carried out by addition of NaCl to a final concentration of 35 mM, Trizma-HCl, pH 8.0 to 6.5 mM, and equimolar primer with respect to template (3 μM, 5′-Alexa Fluor 546-CTGACTGCATCTAGACGTGACTGA-3′), incubation for 5 minutes at 95°C, followed by slow cooling to room temperature over ∼30 minutes. Annealed primer/template was desalted using a Bio-Rad Econo-Pac 10DG column (Bio-Rad, Hercules, CA, USA), equilibrated with 30 ml of 10 mM Tris-HCl, pH 8.0 DNA concentrations of the eluted fractions were determined by measuring absorbance at 260 nm.

**DNA polymerase generation.** A mutant form of φ 29 DNA polymerase, φ29^N62D^, exhibiting reduced 3′-5′ exonuclease activity while maintaining essentially identical polymerizing properties,(1, 2) was cloned into pET41 (Invitrogen, Carlsbad, CA, USA), providing N-terminal Histidine (His) and GST tags for purification. The enzyme was overproduced in *E. coli*. Cells were disrupted using lysozyme (chicken egg white, Sigma-Aldrich, St. Louis, MO, USA) in buffer B (50 mM Tris-HCl, pH 7.5, 7 mM 2-mercaptoethanol, 5% glycerol) containing 0.2 M NaCl; and sonication. DNA was degraded by DNase I (bovine pancreas, Sigma-Aldrich). Cell debris was removed by centrifugation for 30 minutes at 15,000 g. Supernatant was adjusted to 0.5 M NaCl for purification on a 1 ml HisTrap FF column (Ni-resin, GE Healthcare, Piscataway, NJ, USA) equilibrated with buffer B containing 0.5 M NaCl. His-tagged polymerase retained on the HisTrap column was washed with at least 50 column volumes, first using buffer B containing 1 M NaCl, 0.2% Tween-20 and 20 mM imidazole, followed by buffer B containing 0.5 M NaCl and 50 mM imidazole. The polymerase was eluted with buffer B containing 300 mM imidazole. The pooled “HisTrap” fraction was adjusted to 0.2 M NaCl using buffer C (50 mM Tris-HCl, pH 7.5, 1 mM EDTA, 7 mM 2-mercaptoethanol, 5% glycerol). The sample was loaded to a Heparin-Sepharose CL-6B (10 ml, GE Healthcare) equilibrated in buffer C. Polymerase was eluted with buffer C and a gradient of 0-1 M NaCl. The pooled “Heparin” fractions were concentrated using Centricon YM-50 (Millipore, Billerica, MA, USA), and adjusted to the final storage buffer (50 mM Tris-HCl, pH 7.5, 0.2 M NaCl, 1 mM EDTA, 7 mM 2-mercaptoethanol, 50% glycerol).

Protein fractions were analyzed by SDS-PAGE (10% or 15% acrylamide) in all purification steps. The purified polymerase was at least 97% homogenous. Protein concentration was determined both by measuring the absorbance at 280 nm, and by the Bradford method using known amount of BSA (Bio-Rad).

## References

[1] Braslavsky I. (2003). Sequence information can be obtained from single DNA molecules. Proc. Natl. Acad. Sci. USA.

[2] Levene M. J. (2003). ero-mode waveguides for single-molecule analysis at high concentrations. Science.

[3] Anderson J. P. (2005). Incorporation of reporter-labeled nucleotides by DNA polymerases. Biotechniques.

[4] Augustin M. A. (2001). Progress towards single-molecule sequencing: enzymatic synthesis of nucleotide-specifically labeled DNA. J. Biotechnol.

[5] Brakmann S. (2001). The large fragment of Escherichia coli DNA polymerase I can synthesize DNA exclusively from fluorescently labeled nucleotides. Chembiochem.

[6] Tasara T. (2003). Incorporation of reporter molecule-labeled nucleotides by DNA polymerases. II. High-density labeling of natural DNA. Nucleic Acids Res.

[7] Zhu Z. (1994). Directly labeled DNA probes using fluorescent nucleotides with different length linkers. Nucleic Acids Res.

[8] Ju J. (2006). Four-color DNA sequencing by synthesis using cleavable fluorescent nucleotide reversible terminators. Proc. Natl. Acad. Sci. USA.

[9] Mitra R. D. (2003). Fluorescent in situ sequencing on polymerase colonies. Anal. Biochem..

[10] Mulder B. A. (2005). Nucleotide modification at the gamma-phosphate leads to the improved fidelity of HIV-1 reverse transcriptase. Nucleic Acids Res..

[11] Sood A. (2005). Terminal phosphate-labeled nucleotides with improved substrate properties for homogeneous nucleic acid assays. J. Am. Chem. Soc..

[12] Kumar S. (2005). Terminal phosphate labeled nucleotides: synthesis, applications, and linker effect on incorporation by DNA polymerases. Nucleosides Nucleotides Nucleic Acids.

[13] Knorre D. G. (1976). General Method for synthesis of Atp gamma-derivatives. FEBS Lett..

[14] Kadokura M. (1997). Efficient synthesis of gamma-methyl-capped guanosine 5′-triphosphate as a 5′-terminal unique structure of U6 RNA via a new triphosphate bond formation involving activation of methyl phosphorimidazolidate using ZnCl2 as a catalyst in DMF under anhydrous conditions. Tetrahedron Lett..

[15] Blanco L. (1996). Relating structure to function in phi29 DNA polymerase. J. Biol. Chem..

[16] Esteban J. A. (1993). Fidelity of phi 29 DNA polymerase. Comparison between protein-primed initiation and DNA polymerization. J. Biol. Chem..

[17] Blanco L. (1989). Highly efficient DNA synthesis by the phage phi 29 DNA polymerase. Symmetrical mode of DNA replication. J. Biol. Chem..

[18] Yarbrough L. R. (1978). Synthesis and properties of a new fluorescent analog of ATP: adenosine-5¢triphosphoro-gamma-1-(5-sulfonic acid) napthylamidate. Biochem. Biophys. Res. Commun..

[19] Yoshikawa M. (1967). A novel method for phosphorylation of nucleosides to 5′-nucleotides. Tetrahedron Lett..

[20] Hoard D. E. (1965). Conversion of mono- and oligodeoxyribonucleotides to 5-triphosphates. J. Am. Chem. Soc..

